# microRNA-18a is a genetic marker for the early diagnosis of cerebral injury induced by type 2 diabetes

**DOI:** 10.3892/etm.2014.1996

**Published:** 2014-09-30

**Authors:** GANG MAO, LEI LIU

**Affiliations:** 1 Intensive Care Unit, The Fourth People’s Hospital of Jinan, Jinan, Shandong 250031, P.R. China; 2 Department of Gastroenterology, The Fourth People’s Hospital of Jinan, Jinan, Shandong 250031, P.R. China

**Keywords:** miRNA-18a, cerebral injury induced by type 2 diabetes, hypoxia-inducible factor-1α, genetic markers

## Abstract

The present study aimed to investigate the value of microRNA (miRNA)-18a for the early diagnosis of cerebral injury in patients with type 2 diabetes. Blood samples were collected from patients with type 2 diabetes, admitted to hospital between January and December 2013. The patients were randomly divided into three groups, which included one control and two experimental groups of severely and mildly diabetic patients (33 individuals per group). The levels of biochemical indicators in the serum, including S100 protein, neuron-specific enolase, myelin basic protein and endothelin-1, were determined. The mRNA and protein expression levels of hypoxia-inducible factor (HIF)-1α in the serum were measured by quantitative polymerase chain reaction (qPCR) and western blot analysis, respectively. In addition, the serum expression levels of miRNA-18a were determined by qPCR. The concentrations of the biochemical indicators in the severe diabetes group were significantly higher compared with those from the other two groups. Furthermore, the mRNA and protein expression levels of HIF-1α in the severe diabetes group were significantly upregulated compared with the other groups. However, the levels of miRNA-18a in the severe diabetes group were significantly downregulated compared with the other groups. The present study demonstrated that the elevation of biochemical indicators in the serum and the upregulation of HIF-1α mRNA and protein expression are associated with the downregulation of miRNA-18a. Therefore, miRNA-18a may be a potential genetic marker for the early diagnosis of cerebral injury induced by type 2 diabetes.

## Introduction

Diabetes is a metabolic disorder that is typically characterized by high levels of blood sugar. Prolonged high blood sugar levels may cause macrovascular and microvascular diseases, leading to chronic damage and dysfunction of various tissues, particularly the eyes, kidneys, heart, veins and nerves (1). In patients with type 2 diabetes, insulin sensitivity is usually decreased due to insulin resistance. Although insulin resistance may be partially compensated by increasing the levels of insulin in the blood, insulin secretion remains insufficient to overcome the lesions caused by obesity and high levels of blood sugar. The incidence rate of cerebrovascular disease in patients with diabetes is >50%. Lesions on the vascular intima induced by diabetes are widely hypothesized to result in cerebral artery stenosis, which causes local embolization, as well as cerebral ischemia, hypoxia and neuronal necrosis (2). Thus, vascular lesions have always been an important focus of studies investigating the cerebral complications of diabetes. During the occurrence and development of diabetic cerebral injury, vascular disease may induce changes in the expression levels of numerous regulatory factors; hypoxia-inducible factor-1α (HIF-1α) is one of these regulatory factors (3).

The up- and downregulation of genes may be affected by numerous factors with various mechanisms. The microRNA (miRNA) regulatory pathway is one of the most well-studied mechanisms. miRNA-18a belongs to the miR17-92 gene cluster, and HIF-1α is one of the target genes of miRNA-18a (4). The stable gene expression profiles and specificity of miRNA-18a make it a specific marker for the early diagnosis and treatment of a number of diseases that are associated with HIF-1α. A previous study indicated that upregulation of miRNA-18a downregulates the expression level of HIF-1α in tumor tissues (5). However, to the best of our knowledge, the regulatory effect of HIF-1α in diabetic cerebral injury has not been previously investigated.

In order to provide early diagnostic approaches for cerebral injury induced by type 2 diabetes, the present study investigated the expression levels of miRNA-18a in the blood of patients with type 2 diabetes and the association with cerebral injury.

## Materials and methods

### Subjects

According to the diagnostic criteria for diabetes set by the World Health Organization in 1999 (6), type 2 diabetes patients hospitalized at the Fourth People’s Hospital of Jinan (Jinan, China) between January and December 2013 were enrolled in the study. The patients were divided into a control group of healthy subjects and two experimental groups of patients with severe or mild diabetes. The control group comprised 33 individuals, including 18 males and 15 females, with ages ranging from 30 to 76 years (average age, 51.6 years). In the mild diabetes group, there were 33 individuals, including 16 males and 17 females, with ages ranging from 32 to 75 years (average age, 52.4 years). There were 33 individuals in the severe diabetes group, including 13 males and 20 females, with ages ranging between 35 to 72 years (average age, 53.8 years). Intracranial pressure monitoring, brain oxygen partial pressure monitoring and computed tomography scanning were performed on six patients from the severe diabetes group. Blood samples were collected from all the subjects following approval by the Ethics Committee of The Fourth People’s Hospital of Jinan. Informed consent was obtained from the patients or their families.

### Reagents and instruments

miRcute miRNA isolation, miRcute miRNA first-strand cDNA synthesis and miRcute miRNA qPCR detection kits were purchased from Tiangen Biotech Co., Ltd. (Beijing, China). A quantitative polymerase chain reaction (qPCR) iQ5 Optical System and Image Lab^™^ software were obtained from Bio-Rad Laboratories, Inc. (Hercules, CA, USA). The primary antibodies against HIF-1α were purchased from Abcam (Cambridge, MA, USA).

### Sample collection

Blood samples were collected and divided into equal portions. One portion was sent immediately to the hospital laboratories to determine changes in the biochemical indicators with regard to brain-specific changes, including those of the S100 protein (S100B), neuron-specific enolase (NSE), myelin basic protein (MBP) and endothelin-1 (ET-1). The remaining samples were stored in a refrigerator at −80°C.

### qPCR

Total RNA was extracted from the serum using TRIzol reagent (Invitrogen Life Technologies, Carlsbad, CA, USA). The quality of the extracted RNA was confirmed by a NanoDrop 1000 UV-Vis spectrophotometer (optical density ratio at 260/280 nm; NanoDrop Technologies, Wilmington, DE, USA). The total RNA underwent reverse transcription to acquire cDNA.

The primers used for qPCR were as follows: HIF-1α upstream, 5′-GACAAGCCACCTGAGGAGAG-3′ (381 bp) and downstream, 5′-GTTCGCATCTTGATAAGGCC-3′; and β-actin upstream, 5′-GGCATGGGTCAGAAGGATTCC-3′ (316 bp) and downstream, 5′-ATGTCACGCACGATTTCCCGC-3′. The amplification conditions were as follows: Initial denaturation at 94°C for 2 min, followed by 45 cycles of denaturation at 94°C for 30 sec, annealing at 60°C for 1 min and extension at 68°C for 2 min, with a final elongation at 68°C for 7 min. The 2^−ΔΔCt^ method was used to calculate the ratio of the gray levels of HIF-1α against those of β-actin.

For qPCR analysis of miRNA-18a, the primers used were as follows: miRNA-18a upstream, 5′-GATAGCAGC ACAGAAATATTGGC-3′; U6 snRNA upstream, 5′-GCGCGTCGTGAAGCGTTC-3′; and the universal downstream primer, 5′-GTGCAGGGTCCGAGGT-3′. The amplification conditions were as follows: Initial denaturation at 95°C for 10 min, followed by 40 cycles of denaturation at 95°C for 15 sec, annealing at 60°C for 1 min and extension at 72°C for 2 min, with a final elongation at 72°C for 7 min. The 2^-ΔΔCt^ method was used to calculate the ratio of gray levels, where U6 was used as the internal reference.

### Western blot analysis

Total proteins were extracted from the samples according to the procedures for protein lysis (7). Following the determination of the protein sample concentration using a bicinchoninic acid protein assay kit (Pierce, Rockford, IL, USA), the samples were mixed with sodium dodecyl sulfate polyacrylamide gel electrophoresis loading buffer, prior to boiling for 5 min. Protein samples (20 μg) were loaded onto the gel (10%) for electrophoresis, and electrically transferred onto polyvinylidene difluoride membranes (Millipore, Billerica, MA, USA) charged at a constant 100 V in an ice bath for 2 h. The membrane was blocked with skimmed milk (5%) for 1 h at room temperature. Primary antibodies against HIF-1α (1:2,000; rabbit anti-human polyclonal antibody; Abcam, Cambridge, MA, USA) and the internal reference protein, β-actin (1:5,000; abbit anti-human polyclonal antibody; Abcam), were added prior to incubation overnight at 4°C. Following rinsing with phosphate-buffered saline with Tween 20 three times for 10 min, horseradish peroxidase conjugated goat anti-rabbit immunoglobulin G (IgG; 1:3,000; Abcam) was added prior to incubation at room temperature for 1 h. The samples were subsequently rinsed with phosphate-buffered saline with Tween 20 three times for 10 min. The immunoreactive bands were visualized by enhanced chemiluminescence (Pierce). Image Lab^™^ software was used to acquire images and analyze the signal intensity. The relative expression levels of target proteins were calculated from the ratio of the gray levels of the target protein bands against those of the β-actin bands.

### Statistical analysis

Results were analyzed using SPSS 18.0 software (SPSS, Inc., Chicago, IL, USA), and are expressed as the mean ± standard deviation. All data were subjected to a normality test. Multiple sets of measurements were analyzed using one-way analysis of variance. To determine the homogeneity of variance, Fisher’s least significant difference and the Student-Newman-Keuls tests were used. To determine the heterogeneity of variance, Tamhane’s T2 or Dunnett’s T3 tests were used. P<0.05 was considered to indicate a statistically significant difference; P<0.01 was considered to indicate a highly statistically significant difference.

## Results

### Mild diabetes leads to slight cerebral injury, while severe diabetes causes severe cerebral injury, according to the levels of biochemical indicators

To compare the biochemical indicators among the control, mild diabetes and severe diabetes groups, the concentrations of S100B, NSE, MBP and ET-1 were measured. The levels of NSE and MBP in the mild diabetes group were significantly higher compared with those in the control group (P<0.05). In the severe diabetes group, the levels of S100B, NSE, MBP and ET-1 were all significantly higher compared with those in the control group (P<0.01). In addition, the NSE concentration was significantly higher in the severe diabetes group when compared with the mild diabetes group (P<0.05), while the levels of S100B, MBP and ET-1 exhibited a highly statistically significant difference when comparing the two groups (P<0.01; Table I). Notably, the levels of all the biochemical indicators greatly exceeded their clinically normal reference values (Table I). These observations indicated that mild diabetes led to slight cerebral injury, while severe diabetes caused severe cerebral injury.

### HIF-1α mRNA and protein expression markedly increase in patients with severe diabetes, while only HIF-1α protein expression increases in mild diabetic patients

**Figure 1:**
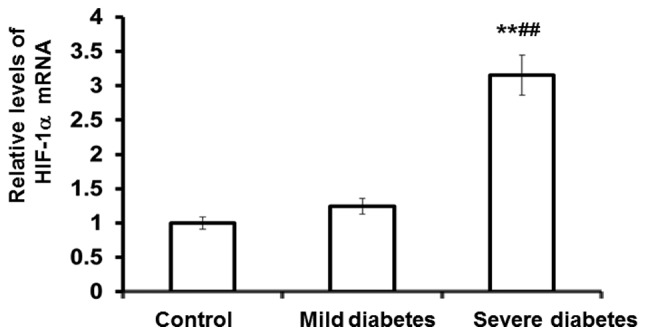
mRNA expression levels of HIF-1α in the control, mild diabetes and severe diabetes groups, as measured by quantitative polymerase chain reaction. Data were normalized against those in the control group and are expressed as the mean ± standard deviation. ^**^P<0.01, vs. control group; ^##^P<0.01, vs. mild diabetes group. HIF, hypoxia-inducible factor.

**Figure 2:**
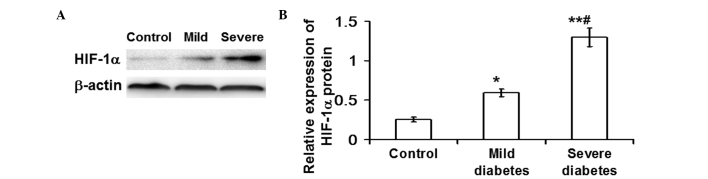
(A) Western blot analysis and (B) quantification of the relative protein expression levels of HIF-1α in the control, mild diabetes and severe diabetes groups. β-actin was used as an internal reference. Data are expressed as mean ± standard deviation. ^*^P<0.05 and ^**^P<0.01, vs. control group; ^#^P<0.05, vs. mild diabetes group. HIF, hypoxia-inducible factor.

To determine the mRNA and protein expression levels of HIF-1α in the control, mild diabetes and severe diabetes groups, qPCR and western blot analysis were performed. The qPCR results revealed that the mRNA expression levels of HIF-1α in the severe diabetes group were significantly elevated compared with those in the control and mild diabetes groups (P<0.01; Fig. 1). In addition, the western blot analysis results demonstrated that the protein expression levels of HIF-1α in the severe diabetes group were significantly elevated compared with the control (P<0.01) and mild diabetes groups (P<0.05). Furthermore, the protein expression of HIF-1α in the mild diabetes group was significantly higher compared with the control group (P<0.05; Fig. 2). These observations indicated that the mRNA and protein expression levels of HIF-1α were markedly increased in patients with severe diabetes, whereas for the mild diabetes patients, an increase in HIF-1α protein expression was observed, but not mRNA expression.

### Expression levels of miRNA-18a are downregulated by severe diabetes

**Figure 3:**
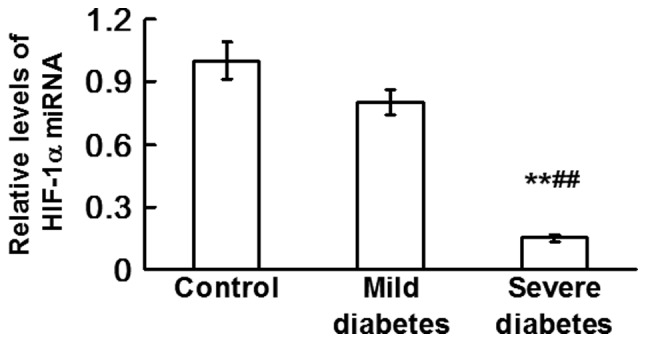
Relative expression levels of miRNA-18a in the control, mild diabetes and severe diabetes groups, as measured by quantitative polymerase chain reaction. Data were normalized against those in the control group and are expressed as the mean ± standard deviation. ^**^P<0.01, vs. control group; ^##^P<0.01, vs. mild diabetes group. HIF, hypoxia-inducible factor; miRNA, microRNA.

qPCR was used to determine the levels of miRNA-18a in the control, mild diabetes and severe diabetes groups. The results revealed that the levels of miRNA-18a in the severe diabetes group were significantly lower compared with those in the control and mild diabetes groups (P<0.01; Fig. 3). This observation indicated that the levels of miRNA-18a were downregulated by severe diabetes, in contrast to the upregulation of HIF-1α mRNA and protein expression.

## Discussion

Cerebral injury, such as brain thrombosis, brain infarction, secondary epilepsy and dementia, is one of the most severe complications of diabetes. However, the pathogenesis of diabetes-induced cerebral injury remains unclear (8). There are various methods of detecting cerebral injury in current clinical practice, including functional tests and biochemical detection. However, clinical application of the functional tests is limited due to patient compliance and affordability. Therefore, detection by biochemical indicators has become the common method for the evaluation of cerebral injury.

Biochemical indicators that reliably reflect brain-specific changes include S100B (9), NSE (10), MBP (11) and ET (12). In the present study, these biochemical indicators were used to evaluate the degree of cerebral injury in patients with diabetes. The data revealed that the levels of biochemical indicators in patients with severe diabetes were distinct from those in the control group, and were markedly higher than the clinically normal reference values, indicating that cerebral injury had already occurred in the patients. Although only two biochemical indicators in the patients with mild diabetes were significantly different from individuals in the control group, all the biochemical indicators in the mild diabetes group were close to or only slightly higher than the clinically normal reference values, indicating that the patients may have had slight cerebral injury that was alleviated through self-regulation or other reasons. The clinical presentations of the patients concurred with the current observations. The majority of the patients with severe diabetes experienced dizziness, tinnitus, sleepiness, transient amnesia and sleep disturbances; however, considerably fewer patients in the other two groups exhibited these presentations. Intracranial pressure monitoring, brain oxygen partial pressure monitoring and computed tomography scanning in several patients revealed increased intracranial pressure, lower oxygen partial pressure and slight thrombus and stenosis in the intracranial veins.

In an environment of local oxygen deficiency or tumor growth, HIF-1α, as an important transcription factor for the regulation of oxygen homeostasis, is upregulated (13). The mechanisms underlying this compensatory and pathological procedure promote angiogenesis, increase the blood supply, improve the situation of oxygen deficiency or meet the requirements for the growth and proliferation of tumors. Angiopathies induced by diabetes, including thrombus and infarction, may cause blood and oxygen deficiency in the whole body, resulting in the upregulation of HIF-1α. Since brain samples are valuable and hard to retrieve from living patients, the detection of HIF-1α expression in the brain tissue is difficult. However, when the severity of cerebral injury induced by diabetes reaches a certain level, HIF-1α enters the blood circulation from the brain. Therefore, the levels of HIF-1α detected in the blood may indirectly reflect the levels of HIF-1α in the brain. In the present study, the mRNA and protein expression levels of HIF-1α in patients with severe diabetes were significantly higher compared with those in the other two groups, indicating that local hypoxia may exist in the brains of severely diabetic patients. Thus, the values of the biochemical indicators for brain-specific changes deviated from the clinically normal reference ranges. As HIF-1α expression increased, the deviation was augmented.

In previous tumor studies (14–17), the upregulation of miRNA-18a was found to downregulate the levels of HIF-1α, inhibiting angiogenesis in tumor cells. In the current study, the expression of HIF-1α was enhanced in the blood of diabetes patients, while the levels of miRNA-18a were downregulated; thus, the results were in accordance with the previous studies. Furthermore, the biochemical indicators in the blood, which reflect brain-specific changes, were also found to be associated with HIF-1α expression.

In conclusion, the levels of the biochemical indicators, HIF-1α expression and miRNA-18a have a fixed association. Changes in the levels of miRNA-18a in the blood indirectly reflect the status of cerebral injury and may be significant in the diagnosis of cerebral injury. However, the present study has certain limitations, including the small sample size, regional differences of the patients, additional cerebral injury factors other than HIF-1α (18–20) and the numerous other miRNAs that regulate these factors. However, miRNA-18a remains indicative of cerebral injury and may provide novel insights for the prevention and treatment of diabetes-induced cerebral injury.

## Figures and Tables

**Table I:** Biochemical indicators for brain-specific changes.

Parameter	S100B (μg/l)	NSE (U/ml)	MBP (ng/ml)	ET-1 (ng/l)
Control	0.04±0.02	9.18±5.23	1.14±0.96	46.1±5.58
Mild diabetes	0.06±0.03	12.08±7.16^a^	1.98±1.03^a^	48.8±7.71
Severe diabetes	0.51±0.16^b^,^d^	16.22±8.18^b^,^c^	6.74±3.59^b^,^d^	59.08±10.66^b^,^d^
Normal reference values	<0.05	<12.5	2.28±1.65	50.8±7.58

a P<0.05 and

b P<0.01, vs. control group;

c P<0.05 and

d P<0.01, vs. mild diabetes group.

S100B, S100 protein; NSE, neuron-specific enolase; MBP, myelin basic protein; ET-1, endothelin-1.
